# Supplementary material for: Rheumatologic Perspectives on Sarcoidosis: Predicting Sarcoidosis-Associated Arthritis Through Comprehensive Clinical and Laboratory Assessment
Source: J Clin Med. 2024 Dec 12;13(24):7563. doi: 10.3390/jcm13247563 (PMC11728293; doi:10.3390/jcm13247563)
Supplement: Supplementary file 1 [file jcm-13-07563-s001.zip › jcm-3283893-supplementary.pdf]

Table S1: Logistic regression analysis of factors influencing the development of arthritis in sarcoidosis patients

	Univariable analysis			Multivariable analysis		
	OR	95% CI	*p* value*	OR	95% CI	*p* value*
**Gender, F/M (n)**	2.068	0.791–5.411	0.139			
**Age, mean ± SD**	1.023	0.991–1.056	0.167			
**Sarcoidosis disease duration (months), mean ± SD**	1.003	0.998–1.010	0.207			
**Age at diagnosis (years), mean ± SD**	1.016	0.982–1.051	0.357			
**Comorbidity, % (n)**	1.517	0.729–3.159	0.265			
**DM, % (n)**	2.727	1.115–6.668	***0.028***	4.805	1.025–22.518	***0.046***
**HT, % (n)**	1.895	0.880–4.080	0.102			
**Asthma, % (n)**	1.273	0.440–3.685	0.657			
**COPD, % (n)**						
**HL, % (n)**	1.324	0.367–4.771	0.668			
**Hypothyroidi**	0.744	0.144–3.837	0.724			
**Erythema nodosum, % (n)**	1.818	0.784–4.217	0.164			
**Eye, % (n)**	0.488	0.154–1.542	0.222			
**Peripheral lymph node, % (n)**	2.541	0.888–7.269	0.082			
**Splenomegaly, % (n)**	1.750	0.375–8.163	0.476			
**Liver, % (n)**	1.535	0.248–9.517	0.645			
**Neurological, % (n)**	1.136	0.100–12.863	0.918			
**CRP (mg/dL), median (IQR)**	0.990	0.956–1.025	0.572			
**ESR (mm/hour), median (IQR)**	1.004	0.992–1.017	0.516			
**AST (U/L), median (IQR)**	1.021	0.992–1.050	0.158			
**ALT (U/L), median (IQR)**	1.034	1.004–1.065	***0.027***			
**ALP (U/L), median (IQR)**	1.004	0.998–1.010	0.232			

**GGT, (U/L), median (IQR)**	1.010	1.001–1.019	***0.025***	1.042	0.983–1.104	0.163
**ACE (U/L), median (IQR)**	0.999	0.992–1.006	0.803			
**Calcium (mg/dL), mean ± SD (min., max.)**	0.987	0.558–1.745	0.965			
**Phosphore (mg/dL), mean ± SD (min., max.)**	0.789	0.446–1.395	0.415			
**Parathormone (ng/L), mean ± SD (min., max.)**	1.009	0.998–1.021	0.116			
**25-hydroxyvitamin D (μg/L), median (IQR)**	0.937	0.885–0.992	***0.025***	0.914	0.841–0.993	***0.034***
**RF positivity (n=112)**	0.537	0.058–4.986	0.584			
**ACPA positivity (n = 78)**	1.065	0.182–6.242	0.940			
**ANA positivity (n = 117)**	1.264	0.561– 2.849	0.582			
**FEV1 (%)**	0.989	0.970–1.009	0.293			
**FVC (%)**	0.986	0.968–1.005	0.137			
**DLCO Adj (mL/mmHg/min.)**	0.983	0.963–1.002	0.079			

F/M: female/male; **OR:** odds ratio; **CI:** confidence interval; **SD:** standard deviation; **min.:** minimum; **Max:** maximum; **HT:** hypertension; **DM:** diabetes mellitus; **HL:** hyperlipidemia; **COPD:** chronic obstructive pulmonary disease; **CRP:** C- reactive protein; **ESR:** erythrocyte sedimentation rate 1st hour; **AST:** aspartate aminotransferase; **ALT:** alanine aminotransferase; **ALP:** alkaline phosphatase; **GGT:** gamma-glutamyl transferase; **ACE:** angiotensin-converting enzyme; **RF:** rheumatoid factor; **ACPA:** anti-citrullinated protein antibody; **ANA:** antinuclear antibody; **FVC:** forced vital capacity; **FEV1:** forced expiratory volume in 1 second; **DLCO:** diffusing capacity of the lungs for carbon monoxide; **mL:** milliliter; **%:** percent; **mmHg:** millimeters of mercury; **min.:** minute; *: statistical significance was defined as $p < 0.05$.
